# Angiotensin-Converting Enzyme 2 SNPs as Common Genetic Loci and Optimal Early Identification Genetic Markers for COVID-19

**DOI:** 10.3390/pathogens11080947

**Published:** 2022-08-22

**Authors:** Yan Ma, Qiuyue Li, Jun Chen, Songmei Liu, Shanshan Liu, Xiaomeng He, Yun Ling, Jianghua Zheng, Christopher Corpe, Hongzhou Lu, Jin Wang

**Affiliations:** 1 Shanghai Public Health Clinical Center, Fudan University, Shanghai 201508, China; 2 Center for Gene Diagnosis, Zhongnan Hospital of Wuhan University, Wuhan 430071, China; 3 Department of Laboratory Medicine, Zhoupu Hospital Affiliated to Shanghai University of Medicine & Health Sciences, Shanghai 201318, China; 4 Nutritional Science Department, King’s College London, 150 Stamford Street, Waterloo, London SE1 9NH, UK; 5 National Clinical Research Centre for Infectious Diseases, The Third People’s Hospital of Shenzhen, The Second Affiliated Hospital of Southern University of Science and Technology, Shenzhen 518112, China

**Keywords:** ACE2, SNP, COVID-19, cardiovascular risks

## Abstract

Background: Angiotensin-converting enzyme 2 (ACE2) is implicated as a host cell receptor that causes infection in the pathogenesis of coronavirus disease 2019 (COVID-19), and its genetic polymorphisms in the ACE2 gene may promote cardiovascular disease and systemic inflammatory injury in COVID-19 patients. Hence, the genetic background may potentially explain the broad interindividual variation in disease susceptibility and/or severity. Methods: Genetic susceptibility to COVID-19 was analyzed by examining single-nucleotide polymorphisms (SNPs) of ACE2 in 246 patients with COVID-19 and 210 normal controls using the TaqMan genotyping assay. Results: We demonstrated that the ACE2 SNPs rs4646142, rs6632677, and rs2074192 were associated with COVID-19 (for all, *p*  <  0.05), and the differences in the ACE2 SNPs rs4646142 and rs6632677 were correlated with COVID-19-related systemic inflammatory injury and cardiovascular risk. Specifically, rs4646142 was associated with high-sensitivity C-reactive protein (hs-CRP), prealbumin (PAB), apolipoprotein A (APOA), high-density lipoprotein (HDL), and acid glycoprotein (AGP) levels. Rs6632677 was also associated with elevated CRP, acid glycoprotein (AGP), and haptoglobin (HPT). Conclusions: Our results suggest that the ACE2 SNPs rs4646142 and rs6632677 may be common genetic loci and optimal early identification genetic markers for COVID-19 with cardiovascular risk.

## 1. Introduction

Severe acute respiratory syndrome coronavirus 2 (SARS-CoV-2), the virus that causes coronavirus disease 2019 (COVID-19) via respiratory infection, was first discovered in Wuhan, China, in December 2019. Since then, the virus has become deeply entrenched in many countries and is an enormous threat to global public health and economy. The ongoing COVID-19 outbreak resulted in more than 584 million SARS-CoV-2 infections and over 6.4 million deaths worldwide by 8 August 2022 [1]. Different clinical symptoms depend on the variant of the virus and include fever [2], dry cough, headache [3], sore throat, nausea, breathlessness, and loss of taste or smell [4], while some infected individuals remain asymptomatic [5,6]. Morbidity and mortality caused by COVID-19 are closely associated with past coexisting diseases, especially cardiovascular diseases [7]. Some clinical and laboratory features were associated with COVID-19 severe/critical symptoms [8,9,10,11,12]. C-reactive protein (CRP) is the main acute-phase protein with a rapid and sharp increase in plasma concentration during infection and tissue damage and may be a biomarker for pulmonary exacerbation manifestation and therapeutic response [13]. Acid glycoprotein (AGP) as the main acute-response protein is abnormally increased in patients with acute inflammation, rheumatic malignant tumors, and myocardial infarction [14]. Obviously, CRP and AGP play vital protective roles in the body’s innate immune process [15,16]. Haptoglobin (HPT) is another acute-phase reaction protein that is significantly increased when the body is in an inflammatory state and is related to the severity and prognosis of disease [8]. Lipid profile features and lipid metabolism are associated with COVID-19 [9,10]. High-density lipoprotein (HDL) as a plasma lipoprotein that counteracts atherosclerosis is a protective factor for coronary heart disease. Apolipoprotein A (APOA) is an important component of plasma HDL as the antiatherosclerosis factor. HDL and APOA levels were significantly lower in a group with severe disease, with mortality cases showing the lowest levels [9]. The measured HDL and APOA levels could serve as clinical risk factors for severe COVID-19 infection [11]. Additionally, the prealbumin (PAB) level at hospitalization is an indicator of prognosis for COVID-19 patients [12]. Moreover, human genetic factors may lead to increased transmissibility of SARS-CoV-2 and contribute to the persistent progressive disease observed in some infected people, but these genetic factors are largely unknown [17]. In our previous studies, four mathematical models were constructed to predict SARS-CoV-2 transmission and the effectiveness of eradication strategies [18]. The effective detection rate and detection time of SARS-CoV-2 quantitative real-time reverse transcriptase–polymerase chain reaction (qRT-PCR) analysis based on the sensitivity and specificity of various antibody detection methods were reviewed [19]. We further investigated whether the determinants of disease severity are mainly derived from host factors. Genetic variation of the virus does not appear to exert a significant effect on the outcomes of COVID-19 [20]. However, abundant studies have indicated the possible association of ACE2 single-nucleotide polymorphisms (SNPs) with cardiovascular risk in patients with cardiovascular disease [21,22,23]. These observations suggest how much of the variation in COVID-19 disease severity may be explained by human genetic DNA polymorphism. Hence, early warning of inflammatory infections and cardiovascular disease risk by identifying host genetic DNA polymorphisms, including excessive immune responses to viruses, will greatly promote the development of new prevention and/or treatment strategies for COVID-19.

At present, the understanding of the physicochemical properties of SARS-CoV-2 is mostly derived from research on SARS-CoV and MERS-CoV. Angiotensin-converting enzyme 2 (ACE2) is highly expressed in human pneumocytes, intestinal epithelial cells, and endothelial cells [24,25]. Given the importance of the 3D structure of proteins in protein–protein interactions (PPIs), it is not surprising that any process resulting in ACE2 3D structure changes could influence COVID-19 cell entry. Accumulating evidence has shown that the 3D structure of ACE2 might be influenced at both the transcriptional and the posttranscriptional level. ACE2 SNPs can impact on protein function, structure, stability, and abundance [26]. Therefore, the fundamental role of ACE2 in virus infection led to the hypothesis that different complications associated with SARS-CoV-2 infection might be due to different ACE2 SNPs [27]. Since the outbreak of the COVID-19 pandemic, several authors have speculated about the role of ACE and ACE2 gene polymorphisms in disease susceptibility and severity [17,28,29,30]. However, the extent to which the variable response to complications (inflammatory infections and cardiovascular disease risk) related to COVID-19 is influenced by the variability of the hosts’ genetic background is unclear.

Using SNPs for DNA sequence comparisons is common in genetic diversity and evolutionary studies and is especially helpful to identify mutated coronavirus genomes [31]. Recently, ACE2 polymorphisms were analyzed in the human, and it was proposed that ACE2 expression could impact the susceptibility of people to cardiovascular disease, hypertension, dyslipidemia, diabetes, and SARS-CoV-2 infection [32]. For example, 8 ACE2 SNPs (rs2074192, rs233575, rs4240157, rs4646156, rs4646188, rs1978124, rs2048683, and rs879922) were related to type 2 diabetes mellitus (T2D) [22]. Furthermore, these ACE2 SNPs were correlated with diabetes-related cardiovascular complications [22]. The association of ACE2 (rs233575) gene polymorphism with blood pressure was also found in adolescents [33]. More discrepant outcomes for COVID-19 disease are connected with ACE2 polymorphism (rs2074192) in obese, smoking male [33]. In addition, two SNPs (K26R and S331F), which are missense variants, can reduce ACE2 receptor affinity for the spike (S) protein [34]. 

In this study, we speculated that genetic factors in the ACE2 gene were likely to affect the prevalence and mortality of COVID-19. By establishing an SNP genotyping method and collecting literature on ACE2 polymorphisms, we chose rs2074192, rs6632677, rs4646142, rs2048683, and rs4240157, five intronic SNPs in ACE2. Based on different genotyping methods, analysis of clinical indicators was performed in COVID-19 patients. We verified that the pathological degree of COVID-19 was associated with the presence of hypertension and diabetes. Furthermore, our observations illustrated that ACE2 SNPs rs4646142 and rs6632677 may be optimal genetic susceptibility marker for COVID-19-related cardiovascular complications. These factors may be further used in genetic association studies of patients with SARS-CoV-2 infection.

## 2. Materials and Methods

### 2.1. Clinical Subjects and Specimens

A total of 246 whole blood samples were collected from COVID-19 patients who were confirmed to have COVID-19 by qRT-PCR from Shanghai Public Health Clinical Center, Fudan University. A total of 210 whole blood samples were collected from normal subjects in Shanghai Public Health Clinical Center, Fudan University. The demographic and baseline clinical characteristics included smoking, drinking, allergies, surgery, and previous coexisting diseases. All procedures were reviewed and approved by the Ethics Committee of Shanghai Public Health Clinical Center (Registration No: 2020-S216-01), and written informed consent was obtained from all subjects.

### 2.2. Genomic DNA Extraction

Peripheral blood (2 mL) samples obtained from COVID-19 patients and normal subjects were stored in ethylenediaminetetraacetic acid tubes at −20 °C until needed. Genomic DNA (gDNA) was extracted from the peripheral blood samples using the TIANamp Blood DNA Kit (TIANGEN Biotech, Cat#: DP304-02, Beijing, China) according to the manufacturer’s protocol. Finally, gDNA was dissolved in 70 μL of RNA-Free water. gDNA integrity and concentration were assessed at approximately 20-50 ng/μL using Nano Drop ND-2000 (ThermoFisher, Waltham, MA, USA).

### 2.3. Genotyping Assay

Five ACE2 SNPs (rs4646142, rs2048683, rs4240157, rs6632677, rs2074192) (ThermoFisher, Cat#: 4351379, Waltham, MA, USA) were subjected to genotyping by the TaqMan fluorescence probe assay (ThermoFisher, Waltham, MA, USA). In total, 20 ng of gDNA per sample was used for the reaction. We performed PCR in a total reaction volume of 5 μL, including 2.5 μL of 2× Master Mix (ThermoFisher, Cat#: 4440038, Waltham, MA, USA), 0.25 μL of 20× TaqMan^®^ Assay Buffer (ThermoFisher, Cat#: 4351379, Waltham, MA, USA), 10 ng of gDNA, and ddH_2_O. The reaction was initiated and kept at 95 °C for 10 min, followed by 40 cycles at 95 °C (15 s) and 60 °C (1 min), using the LightCycler 480 II Instrument (Roche Molecular Systems, Inc., Basel, Switzerland.).

### 2.4. Statistical Analysis

Allele frequencies were calculated following genotyping. Categorical variables are presented as numbers (%), and continuous variables are presented as the median. Categorical variables were compared using a chi-square-test, and continuous variables were compared using one-way analysis of variance. The odds ratio (OR) between the control genotype and SARS-CoV-2 risk genotype for each ACE2 SNP among categorical variables was evaluated using binary logistic regression with a 95% confidence interval. A *p* value less than 0.05 was considered statistically significant. All probabilities are two-tailed.

## 3. Results 

### 3.1. Characteristics of the Study Participants

Among 246 patients who had a laboratory-confirmed SARS-CoV-2 infection, there were 42 cases with mild disease, 184 cases with common disease, 13 cases with critical disease, and 7 cases with severe COVID-19 (Table 1), which were classified according to the latest WHO regulations [35]. The correlations between COVID-19 severity degree and baseline clinical characteristics are shown in Table 1. We found that the proportion of older patients (≥60 years old) with critical and severe COVID-19 was higher than that of patients with common/mild symptoms. The prevalence of major coexisting illnesses such as hypertension (*p* = 0.026) and heart disease (*p* < 0.001) was also more likely in these patients with COVID-19. Interestingly, a lower percentage of patients with drug allergies was discovered among patients with serious, critical disease and severe COVID-19. From the disease origin perspective, COVID-19 disease types were not significantly different in inland and oversea patients (*p* = 0.722). The most-reported symptoms of COVID-19 were fever (80.1%) and dry cough (46.3%); the details are shown in Table S1. Furthermore, the most common initial therapies were oxygen and interferon treatments (Table S1).

### 3.2. Association of ACE2 SNPs with COVID-19

**Figure 1:**
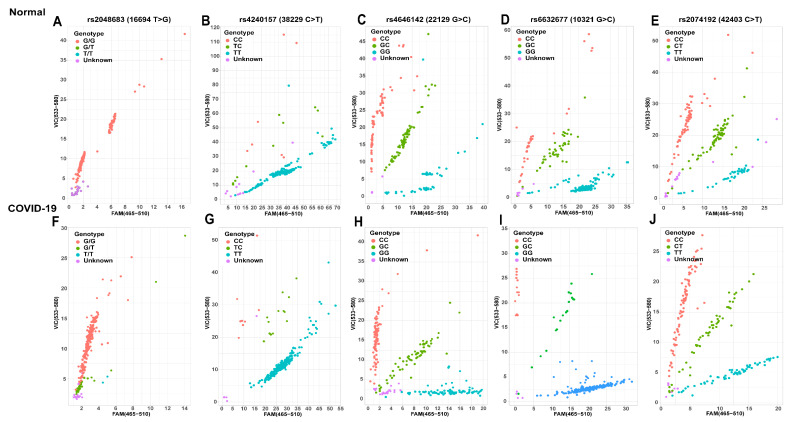
Fluorescence scatter diagrams of ACE2 SNPs in normal controls (**A**–**E**) and COVID-19 patients (**F**–**J**). (**A**,**F**) rs2048683; (**B**,**G**) rs4240157; (**C**,**H**) rs4646142; (**D**,**I**) rs6632677; (**E**,**J**) rs2074192.

To determine further relationships between the five ACE2 polymorphisms and COVID-19, we used the LightCycler^®^480 II system for TaqMan ^®^SNP Genotyping Assays and the LightCycler^®^480SW1.5. software (version 1.5.0.39, Roche, Basel, Switzerland) for data analysis (Figure 1). In rs2048683 (T > G) genotyping (Figure 1A,F), the FAM fluorescently labeled probe targeted the base T, and the VIC fluorescently labeled probe targeted the base G. The prediction analysis of the rs2048683 locus of 210 normal subjects (Figure 1A) in peripheral blood was as follows: TT genotype, 0 cases, TG genotype, 0 cases, GG genotype, 192 cases (scatter plots in red color); 18 cases were not classified into different genotypes (blue). The predictive analysis of the locus in 246 patients with COVID-19 (Figure 1F) provided the following results: TT genotype, 2 cases (blue), TG genotype, 26 cases (green), GG genotype, 198 cases (red); 20 cases were not classified into different genotypes (purple). In rs4240157 (C > T) genotyping (Figure 1B,G), the FAM fluorescently labeled probe targeted the base T, and the VIC fluorescently labeled probe targeted the base C. The prediction analysis of the rs4240157 locus of 210 normal subjects (Figure 1B) in peripheral blood was as follows: CC genotype, 7 cases (red), CT genotype, 11 cases (green), TT genotype, 181 cases (blue); 11 cases were not classified into different genotypes (purple). The predictive analysis of the locus in 246 patients with COVID-19 (Figure 1G) showed: CC genotype, 8 cases (red), CT genotype, 14 cases (green), TT genotype, 220 cases (blue); 4 cases were not classified into different genotypes (purple). In rs4646142 (G > C) genotyping (Figure 1C,H), the FAM fluorescently labeled probe targeted the base G, and the VIC fluorescently labeled probe targeted the base C. The prediction analysis of the rs4646142 locus in 210 normal subjects (Figure 1C) in peripheral blood revealed: CC genotype, 81 cases (red), GC genotype, 73 cases (green), GG genotype, 53 cases (blue); 3 cases were not classified into different genotypes (purple). The predictive analysis of the locus in 246 patients with COVID-19 (Figure 1H) showed: CC genotype, 89 cases (red), GC genotype, 60 cases (green), GG genotype, 77 cases (blue); 20 cases were not classified into different genotypes (purple). In rs6632677 (G > C) genotyping (Figure 1D,I), the FAM fluorescently labeled probe targeted the base G, and the VIC fluorescently labeled probe targeted the base C. The prediction analysis of the rs6632677 locus in 210 normal subjects (Figure 1D) in peripheral blood revealed: CC genotype, 37 cases (red), GC genotype, 43 cases (green), GG genotype, 122 cases (blue); 8 cases were not classified into different genotypes (purple).The predictive analysis of the locus in 246 patients with COVID-19 (Figure 1I) showed: 17 CC genotype cases (red), 19 GC genotype cases (green), 206 GG genotype cases (blue); 4 cases were not classified into different genotypes (purple). In rs2074192 (C > T) genotyping (Figure 1E,J), the FAM fluorescent labeled probe targeted the base T, and the VIC fluorescent labeled probe targeted the base C. The prediction analysis of the rs2074192 locus in 210 normal subjects (Figure 1E) in peripheral blood showed: CC genotype, 84 cases (red), CT genotype, 69 cases (green), TT genotype, 38 cases (blue); 19 cases were not classified into different genotypes (purple). The predictive analysis of the locus of 246 patients with COVID-19 (Figure 1J) revealed: CC genotype, 103 cases (red), CT genotype, 60 cases (green), TT genotype, 77 cases (blue); 6 cases were not classified into different genotypes (purple).

Next, we compared the genotype/allele frequencies between COVID-19 patients and normal controls. The genotype/allele frequencies for ACE2 SNPs rs2074192, rs6632677, rs4646142, rs2048683, and rs4240157 are listed in Table 2. Since the number of genotypes TT and GT of rs2024683 appeared to be zero in the normal group, data statistics could not be conducted, and rs2024683 was eliminated. Based on chi-square-test multiple inheritance models, the ACE2 SNP rs4240157 genotype frequency was not statistically significant different from that of the control groups. The dominant (OR = 0.27, 95% CI: 0.17–0.42, *p* < 0.001), recessive (OR = 0.34, 95% CI: 0.19–0.60, *p* < 0.001), overdominant (OR = 3.17, 95% CI: 1.80–5.67, *p* < 0.001) models showed that rs6632677 was significantly associated with the prevalence of COVID-19, and the recessive (OR = 1.90, 95% CI: 1.21–2.94, *p* = 0.005), overdominant (OR = 1.70, 95% CI: 1.1–2.55, *p* = 0.015) models showed that rs2074192 was significantly related to COVID-19. For rs4646142, there were also significant differences between the GC and GG genotypes (OR = 0.57, 95% CI: 0.35–0.93, *p* = 0.026) and between the CC and GG genotypes (OR = 0.31, 95% CI: 0.20–0.47, *p* < 0.001). These results suggest that ACE2 SNPs rs4646142, rs6632677, and rs2074192 were different between normal subjects and COVID-19 patients.

### 3.3. Association of ACE2 SNPs with Clinical Indicators

**Figure 2:**
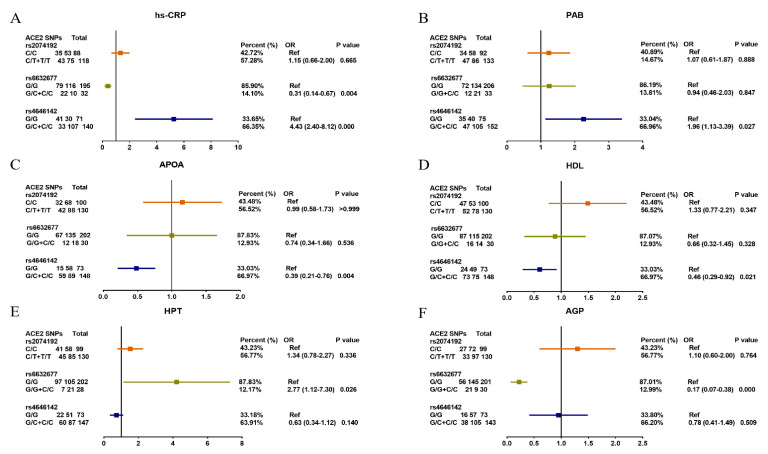
Association of diseases risk-related ACE2 SNPs with clinical biochemical criteria in the study participants. (**A**) High-sensitive C-reactive protein (hs-CRP); (**B**) Prealbumin (PAB); (**C**) Apolipoprotein A (APOA); (**D**) High-density lipoprotein (HDL); (**E**) Haptoglobin (HPT); (**F**) Acid glycoprotein (AGP).

Finally, we evaluated the correlation between ACE2 SNPs and several clinical biochemical markers including hs-CRP, PAB, APOA, HDL, HPT, and AGP among which hs-CRP, PAB, HPT, and AGP are considered acute-phase reactive proteins (APRP). APRP can be identified and monitored with some laboratory tests [36]. These proteins are generally used as markers of inflammation, with a certain tracking and detection accuracy when determining COVID-19 severity and mortality [37]. Additionally, CRP and AGP play a vital protective role in the body’s innate immune process [15,16]. As shown in Figure 2A,F, COVID-19-related ACE2 SNP rs4646142 was associated with increased CRP (*p* < 0.001) level, and rs6632677 could also be associated with increased CRP (*p* = 0.004) and AGP (*p* < 0.001) levels. The ACE2 SNP rs4646142 was correlated with abnormalities of APOA (*p* = 0.004) and HDL (*p* = 0.021) (Figure 2C,D). In Figure 2B, the ACE2 SNP rs4646142 appears significantly associated with prealbumin (PAB) (*p* = 0.029), and PAB was used as a sensitive nutritional protein indicator. In Figure 2E, the ACE2 SNP rs6632677 was further associated with HPT (*p* = 0.026). Notably, the ACE2 SNP rs2074192 was not correlated with any clinical indicators.

## 4. Discussion

Notably, different responses to SARS-CoV-2 infection in different populations raise the possibility that different SNPs profiles might be responsible for the increased risk of acute inflammation, cardiovascular disease, hypertension, diabetes, and stroke in COVID-19 patients. The ACE2 SNP rs2074192 was reported as a risk factor for hypertension in adult obese males [12]. Rs2074192 is related to type 2 diabetes mellitus and cardiovascular disease [22]. Rs4240157, rs6632677, and rs2048683 are also potential genetic susceptibility markers for cardiovascular disease and COVID-19 [38,39], which suggests that these ACE2 SNPs may be biologically relevant to COVID-19. Thus, five ACE2 SNPs (rs4646142, rs2048683, rs4240157, rs6632677, rs2074192) were selected for evaluation as risk factors for COVID-19. In this study, we found that ACE2 SNPs rs4646142, rs6632677, and rs2074192 were associated with COVID-19 following SARS-CoV-2 infection. To the best of our knowledge, this is the first genetic study to analyze if rs4646142, rs6632677, and rs2074192 may increase the odds of COVID-19 infection in the presence of previous coexisting diseases in Chinese individuals. We also found that the ACE2 SNP rs6632677 was correlated with COVID-19 severity [30]. Our findings indicate a prominent role of ACE2 polymorphisms in the pathogenesis of COVID-19.

ACE2 is a transmembrane protein and the main entry point for some coronaviruses into cells, including SARS-CoV, MERS-CoV, and SARS-CoV-2 [40]. ACE2 expression may be related to a high number of membrane-bound viral binding sites, which causes the vulnerability to infection in their carriers. After the receptor binding domain (RBD) in the S1 domain of SARS-CoV and SARS-CoV-2 viral spike proteins binds to the extracellular region of ACE2, spikes may be cleaved by TMPRSS2 on the cell surface, promoting the fusion of the outer membrane of the virus with the host cell membrane and allowing the virus to enter the cytoplasm [41]. Previous studies have reported that among patients with COVID-19 and coexisting hypertension, mortality was lower when ACE inhibitors or angiotensin II receptor blockers (ARBs) were administered than when they were not provided [42]. One study put forward a hypothesis on the role of renin–angiotensin system (RAS) pathway genes including ACE2 (rs2285666, rs1978124, rs714205) in COVID-19 prognosis, suggesting that inherited genetic predispositions could forecast the degree of severity of COVID-19 [43]. In addition, the rs2285666 allele (T or A) was significantly positively correlated with lower infection and mortality among the Indian population [44]. Three ACE2 SNPs, i.e., rs146598386, rs73195521, and rs755766792, were shown to impact the outcome of COVID-19 in a cohort of Russian patients [45]. Nine other ACE2 variants (S19P, I21V, E23K, K26R, T27A, N64K, T92I, Q102P, and H378R) were also predicted to increase the susceptibility to COVID-19 in over 290,000 samples [46], although some ACE2 variants such as rs73635825 and rs143936283 showed completely different effects in different independent studies [47,48,49]. 

ACE2 SNPs are mainly related to human cardiovascular disease, hypertension, dyslipidemia, left myocardial hypertrophy, diabetes, stroke, and retinopathy (Table S2). Through the sequence of UCSC hg19 (exome) and the reference gene homo sapiens angiotensin-converting enzyme 2 (NG_012575.1:6110-46037) on chromosome X, five ACE2 SNPs (rs4646142 G > A or C, rs2048683 T > A or G, rs4240157 C > G or T, rs6632677 G > C, rs2074192 C > T) known to be associated with human cardiovascular diseases and diabetes were examined. Notably, SARS-CoV-2 was most closely associated with bat coronaviruses, because of a 100% amino acid resemblance to bat SL-CoVZC45 in the E proteins and nsp7 [50]. Interestingly, the sequences of rs4646142, rs2048683, rs4240157, rs6632677, and rs2074192 are completely consistent with those of bat ACE2 SNPs, while the SNPs in snakes appear significantly different (Figure S1), suggesting that these SNPs may be associated with SARS-CoV-2 infection. Thus, we further hypothesize that the ACE2 variants rs2074192 and rs6632677 could modify the disease outcome. 

In this study, we investigated possible associations between ACE2 polymorphisms and COVID-19 and demonstrated that the ACE2 SNPs rs4646142, rs6632677, and rs2074192 might be correlated with susceptibility to COVID-19-related cardiovascular risk and acute inflammatory infection. Why exactly do these SNPs impact the susceptibility to COVID-19? We find that rs4646142, rs6632677, and rs2074192 are located in intron regions and cannot encode amino acids. This may be related to the adjustment mode of the intron sequence. A previous study demonstrated that the SNP rs13438494 (intron 24 of PCLO gene) could alter the splicing efficiency by creating or disrupting a splicing motif and ultimately resulted in bipolar disorder in affected people [51]. This implies that the regulation mode of SNPs in the introns of the ACE2 gene also undergo abnormal splicing, which causes the actual mRNA sequence to contain some extra or whole introns or lack some or whole exons, resulting in changes in the amino acid sequence, affecting ACE2 binding to SARS-CoV-2 during virus infection. For instance, in the NS1 protein–RNA interactome, NS1 primarily binds intronic sequences as a multifunctional virulence factor of the influenza A virus, thereby inhibiting cellular processes to accelerate viral gene expression [52]. Human transformer 2 alpha homolog (huTRA2A) can also inhibit mRNA splicing by binding to the intron silencer motif in the NS mRNA of the PR8/H1N1 virus [53]. Some SNPs in tonicity-responsive enhancer binding protein (TonEBP) can impact the transcription process as cis-expressed quantitative trait loci. Meanwhile, these SNPs are associated with an increased risk of T2D, inflammation, and hypertension, indicating that changes in TONEBP expression may be related to these phenotypes [54]. In other words, these SNPs may be able to impact the SARS-Cov-2 infection process by binding to regulatory proteins or RNAs.

## 5. Conclusions

In conclusion, our study showed that the pathological degree of COVID-19 might be associated with hypertension and diabetes, and ACE2 gene variants are associated with the degree of disease severity in COVID-19 patients. Furthermore, the ACE2 SNPs rs4646142 and rs6632677 may be optimal genetic susceptibility markers for COVID-19-related cardiovascular complications, when evaluating the correlations between clinical indicators and SNPs. We demonstrated that the ACE2 SNPs rs4646142 and rs6632677 were significantly associated with increased CRP levels, and rs4646142 was related to abnormalities in APOA, HDL, and PAB, which implies that the correlation between ACE2 SNPs and these lipoproteins is helpful to study the mechanism of injury recovery and novel therapeutic approaches. Overall, our findings support the conclusion that the ACE2 SNPs rs4646142 and rs6632677 correlate with COVID-19-related cardiovascular risk and should be evaluated in large-scale studies.

## Figures and Tables

**Table 1:** Baseline clinical characteristics of patients with COVID-19.

Characteristics	Mild (n = 42)	Common (n = 184)	Severe (n = 7)	Critical (n = 13)	*p* Value
**Age**, median	48	49.5	65	59	0.029
**Distribution**-n (%)					0.001
<60	20 (47.6)	133 (72.3)	2 (28.6)	6 (46.15)	-
≥60	22 (52.4)	51 (27.7)	5 (71.4)	7 (53.85)	-
**Sex**-n (%)					0.704
Male	21 (50.0)	91 (49.5)	5 (71.4)	6 (46.15)	-
Female	20 (47.6)	93 (50.5)	2 (28.6)	7 (53.85)	-
**Smoking**-n (%)	3 (7.14)	12 (6.5)	1 (14.3)	1 (7.69)	0.885
**Drinking**-n (%)	5 (11.9)	15 (8.2)	1 (14.3)	0 (0)	0.541
**Food Allergy**-n (%)	1 (2.38)	0 (0)	0 (0)	0 (0)	-
**Drug Allergy**-n (%)	3 (7.14)	15 (8.2)	0 (0)	1 (7.69)	0.884
**Surgery**-n (%)	2 (4.76)	27 (14.7)	1 (14.3)	2 (15.38)	0.384
**Previous Coexisting Disease**-n (%)					
Hypertension	2 (4.76)	42 (22.8)	3 (42.9)	3 (23.1)	0.026
Diabetes	3 (7.14)	18 (9.8)	2 (28.6)	3(23.1)	0.158
Heart disease	1 (2.38)	7 (3.8)	2 (28.6)	3 (23.1)	<0.001
Pulmonary disease	0 (0)	2 (10.9)	0 (0)	1 (7.7)	0.161
Liver disease	0 (0)	10 (5.4)	0 (0)	1 (7.7)	0.390
Kidney disease	0 (0)	3 (1.6)	0 (0)	0 (0)	
**Origin**-n (%)					0.722
Inland	40 (95.24)	166 (90.2)	6 (85.7)	12 (92.3)	-
Oversea	2 (4.76)	18 (9.8)	1 (14.3)	1 (7.7)	-

The *p* value refers to the chi-square test or ANOVA.

**Table 2:** Genotypes and allele distribution of ACE2 SNPs in human.

SNP	Genotype/Allele Frequency	Normal	COVID-19	Odd Ratio (95% CI)	*p* Value
rs4646142	GG	53 (25.6%)	77 (34.1%)	Ref	
	GC	73 (35.3%)	60 (26.5%)	0.57 (0.35–0.93)	0.026
	CC	81 (39.1%)	89 (39.4%)	0.31 (0.20–0.47)	<0.001
	G	179 (43.0%)	214 (47.3%)	0.85 (0.65–1.11)	0.246
	C	235 (57.0%)	238 (52.7%)		
	CC vs. GC + GG (dominant)			0.99 (0.67–1.46)	>0.999
	CC + GC vs. GG (recessive)			1.50 (0.98–2.27)	0.059
	CC + GG vs. GC (overdominant)			0.66 (0.44–1.00)	0.060
rs20248683	TT	0 (0%)	2 (0.8%)	Ref	
	GT	0 (0%)	26 (11.5%)	0 (to infinity)	>0.999
	GG	192 (100%)	198 (87.6%)	0 (0–2.25)	0.499
	T	0 (0%)	30 (6.6%)	0 (0–0.14)	<0.001
	G	384 (100%)	422 (93.4%)		
	GG vs. GT + TT (dominant)			Na (6.96–Na)	>0.999
	GG + GT vs. TT (recessive)			infinity (0.39–infinity)	0.499
	GT vs. GG + TT (overdominant)			0 (0–0.14)	<0.001
rs4240157	CC	7 (3.5%)	8 (3.3%)	Ref	
	CT	11 (5.5%)	14 (5.8%)	1.11 (0.29–4.15)	>0.999
	TT	181 (91.0%)	220 (90.9%)	1.06 (0.40–3.00)	>0.999
	C	25 (6.0%)	30 (6.2%)	1.01 (0.60–1.75)	>0.999
	T	373 (94.0%)	454 (93.8%)		
	TT vs. CT + CC (dominant)			1.00 (0.52–1.92)	>0.999
	TT + CT vs. CC (recessive)			2.58 (1.11–6.51)	0.053
	CT vs. TT + CC (overdominant)			0.95 (0.43–2.07)	>0.999
rs6632677	GG	122 (60.4%)	206 (85.1%)	Ref	
	GC	43 (21.3%)	19 (7.9%)	0.26 (0.14–0.47)	<0.001
	CC	37 (18.3%)	17 (7.0%)	0.27 (0.15–0.50)	<0.001
	G	287 (71.0%)	431 (89.0%)	1.04 (0.49–2.35)	>0.999
	C	117 (29.0%)	53 (11.0%)		
	GG vs. GC + CC (dominant)			0.27 (0.17–0.42)	<0.001
	GG + GC vs. CC (recessive)			0.34 (0.19–0.60)	<0.001
	GC vs. GG + CC (overdominant)			3.17 (1.80–5.67)	<0.001
rs2074192	CC	84 (44.0%)	103 (42.9%)	Ref	
	CT	69 (36.1%)	60 (25.0%)	0.71 (0.45–1.11)	0.139
	TT	38 (19.9%)	77 (32.1%)	1.65 (1.03–2.65)	0.053
	C	237 (62.0%)	266 (55.4%)	2.33 (1.39–3.87)	0.002
	T	145 (38.0%)	214 (44.6%)		
	CC vs. CT + TT (dominant)			1.04 (0.71–1.53)	0.845
	CC + CT vs. TT (recessive)			1.90 (1.21–2.94)	0.005
	CT vs. CC + TT (overdominant)			1.70 (1.11–2.55)	0.015

*p* value is for the chi-square test, Abbreviation: OR, odds ratio, CI, confidence interval.

## Data Availability

All data generated or analyzed during this study are included in this published article.

## Supplementary Materials

The following supporting information can be downloaded at: https://www.mdpi.com/article/10.3390/pathogens11080947/s1, Figure S1: Blast analysis of ACE2 SNPs; Table S1: Frequency of signs and symptoms according to COVID-19 phenotypes; Table S2: Five ACE2 SNPs in this study are associated with coexisting diseases in previous studies.
