# Supplementary material for: Angiotensin-Converting Enzyme 2 SNPs as Common Genetic Loci and Optimal Early Identification Genetic Markers for COVID-19
Source: Pathogens. 2022 Aug 22;11(8):947. doi: 10.3390/pathogens11080947 (PMC9415427; doi:10.3390/pathogens11080947)
Supplement: Supplementary file 1 [file pathogens-11-00947-s001.zip › pathogens-1764763-supplementary.pdf]

Supplemental information

Angiotensin-converting enzyme 2 SNPs as the common genetic loci and optimal early identification genetic markers for COVID-19

Yan Ma[1, #], Qiuyue Li[1, #], Jun Chen[1, #], Songmei Liu[2, #], Shanshan Liu[1], Xiaomeng He[1], Yun Ling[1], Jianghua Zheng[3], Christopher Corpe[4], Hongzhou Lu[5, *], Jin Wang[1, *]

[1]Shanghai Public Health Clinical Center, Fudan University, Shanghai, China; [2]Center for Gene Diagnosis, Zhongnan Hospital of Wuhan University, Donghu Road 169#, Wuhan 430071, P.R. China; [3]Department of Laboratory Medicine, Zhoupu Hospital Affiliated to Shanghai University of Medicine & Health Sciences, Shanghai, China; [4]King's College London, London, Nutritional Science Department, 150 Stamford Street, Waterloo, London, SE19NH, United Kingdom; [5]National Clinical Research Centre for Infectious Diseases, The Third People’s Hospital of Shenzhen and The Second Affiliated Hospital of Southern University of Science and Technology, Shenzhen 518112, Guangdong Province, China.

Running Title: ACE2 SNPs as the optimal early identification genetic markers for COVID-19

***Correspondence should be addressed to:**

Jin Wang, M.D.& Ph.D.

Scientific Research Center,

Shanghai Public Health Clinical Center

Fudan University

2901 Caolang Road, Jinshan District

Shanghai 201508, China

Ph: 86-21-57036495

Fax: 86-21-57247094

Email: wjincityu@yahoo.com

Hongzhou Lu, M.D.& Ph.D.

National Clinical Research Centre for Infectious Diseases,

The Third People’s Hospital of Shenzhen and The Second Affiliated Hospital of Southern University of Science and Technology,

Shenzhen 518112, Guangdong Province, China.

Tel: +86-18930810088

E-Mail: luhongzhou@fudan.edu.cn

1. Supplemental Figures

1.1. **Supplementary Fig. S1.** The blast analysis of ACE2 SNPs

rs6632677：10321C>G

Homo sapiens. Seq	TGCATTCAAGGTGCTGGCTAGAGCTATGGTCTCT	4232
Myotis brandtii (Brandt's bat).seq	CTGACTGGATGCCAGGTAACCAGCAGCTACACCT	5456
Consensus	a t a g g agc ct	

rs2048683：16694A>T

Homo sapiens. Seq	CCTAAATTAGATGAAATTACCACTTCATAAAGATA	10598
Myotis brandtii (Brandt's bat).seq	AGGAGTCAACAAACTTTTATATAAAGGTAAAGATA	11024
Consensus	a a a tta taaagata	

rs4646142：22129C>T

Homo sapiens. Seq	CCATGGACCCAATAAAGCAACTACACAATCGAGA	16024
Myotis brandtii (Brandt's bat).seq	TTAGGAGAAAAATAAATCTTCCAAAAACCTTGGA	18131
Consensus	a g aataaa c c a a ga	

rs4240157：38229G>C

Homo sapiens. Seq	GTAAATAGTGTTCAGGGGTTTGATTCTGTAATGT	32256
Myotis brandtii (Brandt's bat).seq	GACTATTGCCTTCAGAGGGTTTGAACATTCTAAGG	32997
Consensus	g at g ttcag gggtttga t a g	

rs2074192：42403G>A

Homo sapiens. Seq	CACAAATGAATAAATGCCAACCATTATACATTT	36062
Myotis brandtii (Brandt's bat).seq	AACTCTGGTCGAACTCCTGGTTGAGGGGACAATT	37469
Consensus	ac g aa t c aca tt	

2. Supplemental Tables

2.1. Supplementary Table S1. Frequency of signs and symptoms according to COVID-19 phenotypes.

Signs and symptoms	Mild (n =42)	Common (n =184)	Severe (n =7)	Critical (n =13)	Total (n =246)
Fever	27 (64.3%)	155 (84.2%)	7 (100%)	8 (61.5%)	197 (80.1%)
Dry cough	20 (47.6%)	83 (45.1%)	3 (42.9%)	8 (61.5%)	114 (46.3%)
Expectoration	6 (14.3%)	30 (16.3%)	2 (28.6%)	6 (46.2%)	44 (17.9%)
Dyspnea	0 (0)	7 (3.8%)	1 (14.3%)	3 (23.1%)	11 (4.5%)
Headache	2 (4.8%)	11 (6%)	0 (0)	0 (0)	13 (5.3%)
Dizziness	1 (2.4%)	5 (2.7%)	0 (0)	1 (7.7%)	7 (2.8 %)
Fatigue	4 (9.5%)	7 (3.8%)	0 (0)	4 (30.8%)	15 (6.1%)
Sore/Itchy/dry throat	3 (7.1%)	12 (6.5%)	0 (0)	4 (30.8%)	19 (7.7%)
Nasal congestion	2 (4.8%)	7 (3.8%)	0 (0)	1 (7.7%)	10 (4.1%)
Runny nose	1 (2.4%)	7 (3.8%)	0 (0)	1 (7.7%)	9 (3.7%)
Diarrhea	1 (2.4%)	3 (1.6%)	0 (0)	0 (0)	4 (1.6%)
Myalgia	0 (0)	5 (2.7%)	0 (0)	1 (7.7%)	6 (2.4%)
Chilly	0 (0)	8 (4.3%)	0 (0)	1 (7.7%)	9 (3.7%)
Others symptoms	2 (4.8%)	23 (12.5%)	0 (0)	2 (15.4%)	27 (11.0%)
**Initial therapy**					
Oxygen therapy	2 (4.8%)	56 (30.4%)	7 (100%)	9 (69.2%)	74 (30.1%)
Prezcobix	0 (0)	14 (7.6%)	0 (0)	1 (7.7%)	15 (6.1%)
Arbidol	12 (28.6%)	40 (21.7%)	2 (28.6%)	2 (15.4%)	56 (22.8%)
Interferon	12 (28.6%)	71 (38.6%)	2 (28.6%)	6 (46.2%)	91 (37.0%)
Lopinavir and Ritonavir Tablets	0 (0)	37 (20.1%)	2 (28.6%)	1 (7.7%)	40 (16.3%)
Meropenem	0 (0)	1 (0.5%)	0 (0)	1 (7.7%)	2 (0.8%)
Moxifloxacin	3 (7.1%)	15 (8.1%)	0 (0)	2 (15.4%)	20 (8.1%)
Omeprazole	1 (2.4%)	3 (1.6%)	0 (0)	2 (15.4%)	6 (2.4%)
Vitamin C	0 (0)	15 (8.2%)	1 (14.3%)	1 (7.7%)	17 (6.9%)
Hydroxychloroquine	7 (16.7%)	7 (3.8%)	1 (14.3%)	0 (0)	15 (6.1%)
ShuFengJieDuJiaoNang	3 (7.1%)	10 (5.4%)	1 (14.3%)	0 (0)	14 (5.7%)
Others agents	14 (33.3%)	27 (14.7%)	1 (14.3%)	9 (69.2%)	51 (20.7%)

Signs and symptoms were collected from the electronic medical records for the hospitalized patients.

2.2. Supplementary Table S2. Five ACE2 SNPs in this study are associated with coexisting diseases in the previous studies.

Group	rs2048683	rs2074192	rs4240157	rs6632677	rs4646142
Cardiovascular	+	+	+	-	-
hypertension	+	+	+	+	+
Dyslipidemia	+	+	+	+	+
Left ventricular hypertrophy	-	+	+	+	-
diabetes	+	+	+	-	-
Stroke	-	+	-	-	+
Retinopathy	-	+	-	-	-
Number of articles published	3	10	6	4	1

+ represents literature reported, - represents no literature reported
